# Bayesian Spatial Modeling of Diabetes and Hypertension: Results from the South Africa General Household Survey

**DOI:** 10.3390/ijerph19158886

**Published:** 2022-07-22

**Authors:** Ropo E. Ogunsakin, Themba G. Ginindza

**Affiliations:** 1 Discipline of Public Health Medicine, School of Nursing and Public Health, University of KwaZulu-Natal, Private Bag X54001, Durban 4000, South Africa; ginindza@ukzn.ac.za; 2 Cancer & Infectious Diseases Epidemiology Research Unit (CIDERU), College of Health Sciences, University of KwaZulu-Natal, Private Bag X54001, Durban 4000, South Africa

**Keywords:** Bayesian inference, diabetes, hypertension, spatially varying coefficients, conditional autoregressive

## Abstract

Determining spatial links between disease risk and socio-demographic characteristics is vital in disease management and policymaking. However, data are subject to complexities caused by heterogeneity across host classes and space epidemic processes. This study aims to implement a spatially varying coefficient (SVC) model to account for non-stationarity in the effect of covariates. Using the South Africa general household survey, we study the provincial variation of people living with diabetes and hypertension risk through the SVC model. The people living with diabetes and hypertension risk are modeled using a logistic model that includes spatially unstructured and spatially structured random effects. Spatial smoothness priors for the spatially structured component are employed in modeling, namely, a Gaussian Markov random field (GMRF), a second-order random walk (RW2), and a conditional autoregressive (CAR) model. The SVC model is used to relax the stationarity assumption in which non-linear effects of age are captured through the RW2 and allow the mean effect to vary spatially using a CAR model. Results highlight a non-linear relationship between age and people living with diabetes and hypertension. The SVC models outperform the stationary models. The results suggest significant provincial differences, and the maps provided can guide policymakers in carefully exploiting the available resources for more cost-effective interventions.

## 1. Background

Non-communicable diseases (NCDs) continue to be significant global public health challenges, being responsible for sizeable mortality and morbidity, and their frequency is expanding in low- and middle-income countries [1,2]. Moreover, this has been attributed to increasing life expectancy and globalization of food manufacturing [1,3]. For instance, out of the 57 million total deaths in 2008, statistics indicated that approximately 63% were attributed to NCDs, while annual deaths are anticipated to rise globally [4]. Additionally, in sub-Saharan African (SSA) countries, infectious diseases have been identified as the principal cause of death [5,6,7]. However, due to the treatment intervention of infectious diseases, predominantly HIV, as life expectancy increased, so did the prevalence of NCDs [5]. Other studies have attributed a higher proportion of deaths in SSA to infectious settings and projected that, by 2030, NCDs would cause 46% of deaths [5]. Likewise, published statistics on disease threat estimates in South Africa indicate that NCDs triggered 28% of the total disease threat measured by disability-adjusted life years (DALYs) [8]. This is projected to upsurge significantly over the next few decades if proper measures do not fight the inclination [5]. On the other hand, the menace of these diseases is rising in rural settings of South Africa; they suspiciously affect underprivileged individuals living in urban locations [9]. For example, in the Western Cape, one of the provinces of South Africa, NCDs constitute five of the ten leading causes of death [10].

Moreover, the World Health Organization (WHO) estimates that the threat of the risk of death associated with NCDs is two to three times higher in South Africa than in high-income countries (developed) [3,11]. Meanwhile, the spread of NCDs shows socioeconomic inequalities, with a substantial burden among disadvantaged people in urban settings. With this background, the increasing morbidity and mortality from NCDs have important implications for the distribution of acute and chronic health care services [3]. Moreover, this has financial consequences for households, individuals, and nations [11].

Therefore, predicting NCDs’ progression and geographic spread is vital in public health for resource allocation. With this background, this study focused on modeling hypertension and diabetes, knowing that they are two significant NCDs that significantly contribute to the drain of all cardiovascular diseases (CVDs) and the risk of death. It was established that the epidemiology of hypertension and diabetes divulges various risk factors. In line with this, studies have revealed that socioeconomic factors (such as low levels of educational status and high household wealth index) and demographic factors (such as age and gender—specifically, male) increase the risk of hypertension and diabetes [12,13]. Similarly, lifestyle behaviors (such as smoking and alcohol consumption) and dietary habits also significantly influence the risk of hypertension and diabetes [12,14,15]. Other studies and systematic reviews between 1980 and 2018 have shown an overall estimate of 57% for the frequency of hypertension [16]. Other studies reported a substantial disparity in the frequency of those aware of their hypertension status, at 7 to 56% [17]. At the same time, systematic reviews and meta-analyses have reported that urban residents were at an increased risk of hypertension and diabetes than those in rural settings [18]. Furthermore, another meta-analysis of studies on undiagnosed diabetes [19] indicated that the incidence of diabetes varied between 3.9% and 8.7%, with an overall estimate of 5.4%. As predicted by the International Diabetes Federation, diabetes is expected to increase to 13% by 2030 [20]. 

With this background, a clear understanding of South Africa’s spatial distribution of NCDs, which can bring about accurate disease modeling and mapping in understanding the burden of NCDs across different districts, is needed. However, no spatial analysis of diabetes and hypertension using spatially varying coefficient (SVC) models has been reported in South Africa. The drive of spatial modeling in public health comprises three circles: unfolding current spatial patterns of risk; trying to comprehend the biological apparatuses that lead to disease existence; and estimating what will ensue in the medium- to long-term future (temporal prediction) or in diverse geographical areas (spatial prediction).

Meanwhile, individuals in the same geographical area frequently share beliefs and cultures, which may result in comparable levels of exposure to diseases, such as the one considered in the present study [21,22,23]. Thus, countries with varied cultures and widespread dietary habits are expected to have considerable variations in the prevalence of people living with hypertension and diabetes based on their geographical location [24]. Therefore, understanding the spatial distribution of people living with hypertension and diabetes is vital for measuring end strategy achievements at the regional level.

The success of any health care mediation plan primarily hinges on a wide-ranging and exact consideration of various features that ascertain the existence of diseases and death. Thus, considering the standing of health care in South Africa, the current statistics on the reality of NCDs have been founded mainly on data from hospitals. However, hospital data need to be enhanced by household survey data, because they gather only a subset of infections and have a bias towards more severe outcomes. As such, they may not be regarded as the final appropriate measure when modeling the existence of hypertension, diabetes, and other NCDs for proper plan growth. On the other hand, the household survey remains one valuable source that, combined with additional complementary information, can help to provide the evidence base required for a better understanding of human resources for health and resource allocation. As a surrogate, the demographic survey housed by the South Africa General Household Survey (GHS) collected various information on the health status of the citizens of South Africa. Thus, this study intends to perform spatial modeling on people living with diabetes and hypertension to capture this non-linearity of covariates using the GHS data. The literature shows spatial modeling of NCDs using a standard generalized linear regression model [25,26,27]. Many studies have adopted a standard generalized linear regression model utilized in spatial data analysis, but most of those studies assumed mean and covariance stationarity [26,27]. Mean stationarity suggests a constant association between the outcome of interest and a set of covariates over the region. This assumption is unrealistic because of spatial dependencies and unknown factors that may impact the outcome. Thus, this assumption can only be realistic when the regression coefficients vary across space [28]. Therefore, the problem of non-stationarity can be accommodated by allowing the relationships measured to vary over space through the geographically weighted regression (GWR) model or spatially varying coefficients parameter (SVCP) [29,30].

Most countries implement GHSs to understand specific health problems and determine the prevalence and awareness of different diseases. GHS data are a public instrument to trace and solve many nations’ developmental challenges. The survey data provide ample information on various diseases, and many studies have applied different statistical methods to analyze household survey data. However, most of these methods have been utilized in a manner that may limit interpretations, considering some underlying assumptions. For instance, accounting for spatial variations using Laplace integrated nested approximation (INLA) when modeling NCDs was the central focus of a recent paper by Roy et al. [31]. The study assumed that all of the covariates in the analysis had a linear relationship with the outcome of interest. Borrowing strength from the literature, current research admits that this linear relationship may not hold for all variables. Thus, the present study proposes an approach to relax this stationarity and the linearity assumption considering both the SVC model and RW2. Specifically, this study offers a generalized linear model (GLM) to build the SVC model and compares it with the stationary model. The SVC model is used to relax the stationarity hypothesis, which the previous study failed to address. The non-linear effects of age are captured by a second-order random walk (RW2) and enable covariates to vary in space using the conditional autoregressive (CAR) model. The Bayesian posterior is attained by INLA techniques, a capable substitute for frequently used Markov chain Monte Carlo techniques (MCMC). This paper contributes to the understanding of spatial variations in diabetes and hypertension in South Africa using the SVC model approach based on the INLA technique. Findings from this study can practically benefit the government and decision-makers as both search for an improved understanding and response to the threat posed by NCDs. Similarly, when the covariate predictors are considered based on the spatial model, the modeling and mapping will produce accurate maps useful for health policy.

## 2. Data and Model

### 2.1. Study Area and Data

**Figure 1:**
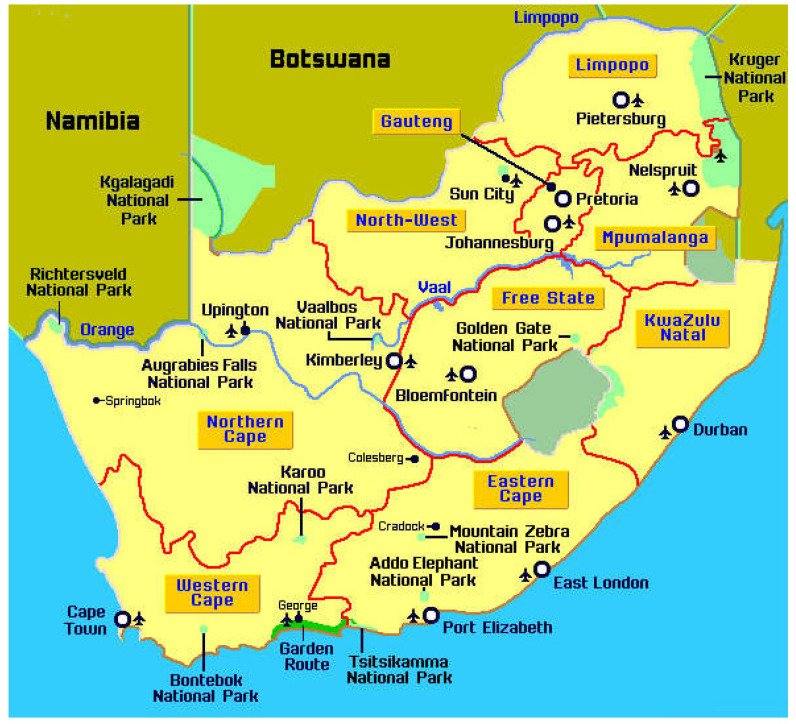
Map of South Africa showing the nine provinces and major cities [34].

The focus of the study was South Africa. There are nine provinces in South Africa: Western Cape (WC), Eastern Cape (EC), Northern Cape (NC), Free State (FS), KwaZulu-Natal (KN), North West (NW), Gauteng (Gau), Mpumalanga (Mp), and Limpopo (Lim). The map of South Africa showing the nine provinces and their major cities is displayed in Figure 1. Data used in the analysis were drawn from the GHS, conducted across the country in 2019. It is collected by Statistics South Africa (Stats SA) annually and made accessible free of charge on its website. Stats SA provides South African data that can be accessed via [32]. The GHS 2019 collection is based on the 2013 Master Sample (MS). This MS is based on information collected during the 2011 census conducted by Stats SA [33]. The MS makes use of a two-stage, stratified design with probability proportional to size (PPS) sampling of primary sampling units (PSUs) from within strata and systematic sampling of dwelling units (DUs) from the sampled PSUs [33]. The country was divided into 103,576 enumeration areas (EAs) before the 2011 census.

The census EAs and the auxiliary information for the EAs were used as the frame units for forming PSUs for the MS. The sample size was composed of 3324 PSUs in the MS, with an expected sample of approximately 33,000 DUs, using the power allocation method. PSUs are enumeration areas (EAs) from the census list with a household count of more than 25, excluding workers’ hostels, convents, and monasteries [33]. The PSUs were sampled in each district using a probability proportional to the number of households in a PSU as calculated in the census. In each PSU, dwelling units were selected using a systematic sampling technique. The MS was designed to be representative at the provincial level and within provinces at metropolitan and nonmetropolitan geographical area levels [33]. Within the metropolitan, the sample is further distributed by geographical type. This implies that, within a metropolitan area, the sample is representative of the different geography types that may exist within that metropolitan area. A stratified design with a PPS selection of PSUs was used in the first stage. In contrast, DU sampling with systematic sampling was employed in the second stage. The survey’s target population consisted of all private households in all nine provinces of South Africa and residents in workers’ hostels. There are 68,986 observations in the 2019 GHS dataset, with 149 variables. Among the 68,986 people interviewed, 15,455 (22.4%) resided in Gauteng, 12,462 (18.1%) were in KZN, and 9279 (13.5%) were from EC; Lim (8192, 11.9%), WC (6051, 8.8%), Mp (5901, 8.6%), NW (4420, 6.4%), FS (4070, 5.9%), and NC (3156, 4.6%). While the 2019 GHS sample included 68,986 individuals aged zero (0) years and older, only persons aged 35 and above were included in the model.

### 2.2. Model Specification and Statistical Analysis

Frequentists and Bayesians extensively use logistic regression models to study the association between covariates and the binary response outcome. Moreover, it has received much consideration in disease mapping to model dichotomous response data in order to describe geographic variation that arises in the data. In the current study, we assumed that $y_{ij}$ represented the binary status of hypertension (hypertensive/non-hypertensive) or diabetes (diabetic/non-diabetic) for an individual i in province *j*: *j* = 1, 2, …, 9. For a randomly selected individual, the probabilistic behavior is typically explained by the univariate Bernoulli probability function—that is, $y_{ij}\sim Bern(p_{ij})$—such that $p_{ij}$ is the probability of being hypertensive (or being diabetic) for the *i*th individual in the *j*th province and, thus, $E\left(Y_{ij}\right)=p_{ij}$ and $Var\left(Y_{ij}\right)=p_{ij}\left(1-p_{ij}\right)$. The GLM approach linked the mean response and the potential *k* predictors ${\left(x_{ij1},\ldots \ldots \ldots \ldots ,x_{ijk}\right)}^{T}$ so that a function $p_{ij}$ was equal to a linear combination of the predictors. The province of the respondent was labeled as $s_{j}\in$ (1, 2, 3, …, 9), where the label matched the labels on the map. The spatial effect of the province $s_{j}$ where the respondent resided was represented by $f_{spatial}\left(s_{j}\right)$. The spatial effect comprised two parts: a structured effect and an unstructured effect. Thus,

$$f_{spatial}\left(s_{j}\right)=f_{str}\left({province}_{j}\right)+f_{unstr}\left({province}_{j}\right)$$

Thus, the following models were formulated:

(1) $$\log it\left(p_{ij}\right)=\beta_{0}+\sum_{m=1}^{k}\beta_{m}x_{ijm}$$

(2) $$\log it\left(p_{ij}\right)=\beta_{0}+\sum_{m=1}^{k}\beta_{m}x_{ijm}+\nu_{j}$$

(3) $$\log it\left(p_{ij}\right)=\beta_{0}+\sum_{m=1}^{k}\beta_{m}x_{ijm}+s_{j}$$

(4) $$\log it\left(p_{ij}\right)=\beta_{0}+\sum_{m=1}^{k}\beta_{m}x_{ijm}+\nu_{j}+s_{j}$$

where $\beta_{0}$, $\nu_{j}$, and $s_{j}$ represent the intercept, spatially unstructured term, and spatially structured term, respectively. Additionally, $\nu_{j}$ accounts for unexplained variability in the model [35], while $s_{j}$ describes the effect of location by assuming that geographically close areas are more similar than distant areas [36,37]. The structured part of the spatial effect was modeled by assigning a Gaussian Markov random field (GMRF) [38,39,40]. The GMRF is a direct generalization of a first-order random walk to two dimensions. In this approach, two regions (provinces), ${province}_{a}$ and ${province}_{b}$, are defined as neighbors if they share a common boundary. Suppose that the index $s\in \left(1,2,\ldots \ldots ,P\right)$ represents the geographically connected regions. The spatial smoothness prior to the function $f_{str}\left(province_{j}\right)$ evaluations using the GMRF is given by

$$f_{str}\left(province_{j}\right)|f_{str}\left(province_{k}\right),k\neq j,\tau_{str}^{2}∼N\left(\frac{\Sigma_{k\in N_{\left(j\right)}}f_{str}\left(province_{k}\right)}{d_{j}},\frac{\tau_{str}^{2}}{d_{j}}\right)$$

where $N_{\left(j\right)}$ and $d_{j}$ are the set and number of adjacent regions *i*, respectively. Therefore, the conditional mean $f_{str}\left(province_{j}\right)$ is an average evaluation of the function $f_{str}\left(province_{j}\right)$ of neighboring districts. On the other hand, $\tau_{str}^{2}$, a spatial variance, measures the amount of spatial heterogeneity [39]. In addition, the unstructured part of the spatial $f_{unstr}\left(province_{j}\right)$ was assigned i.i.d. Gaussian priors. This means that $f_{unstr}\left(province_{j}\right)=a_{j}$, with $a_{j}∼N\left(0,\sigma_{unstr}^{2}\right),j=1,\ldots \ldots ,P$, where *P* denotes the total number of provinces.

### 2.3. Specification of the Spatially Varying Coefficient Model (SVC)

In spatial data analysis, the well-known issue is identifying the nature of the association between variables. However, in many scenarios, a simple model may not describe the association between some sets of variables, sometimes referred to as spatial non-stationarity. Thus, the model must reflect the spatially varying construction within the data to overcome this shortcoming. Based on previous studies, the assumption is that the relationship between the outcome and independent variables is constant throughout the study region [28,41]. However, this assumption may not be realistic for spatial processes, due to the contribution of factors such as altitude and cultures. The two commonly used spatially varying models are the GWR and SVC models. This study focused on the SVC model, a statistical approach developed to relax the spatial stationarity assumption of a regression relationship for spatial data. In addition, the Bayesian spatially varying parameter model was used here to make an inference. The disparity between the present study and other previous studies on NCDs is that our study allowed for coefficients to vary spatially. The specification of the SVC model involves two distinct stages [42]. The starting point consists of the specification of the data distribution conditional on unknown parameters. At the same time, the latter stage entails the specification of the unknown parameters dependent on the other parameters. Based on previous studies [42], the SVCP model is represented as follows:

$$y_{ij}|p_{ij}\sim Ber(p_{ij})$$

$$\psi \left(\eta_{ij}\right)=\log it\left(p_{ij}\right)=X_{ij}^{T}\beta +G_{ij}^{T}\pi$$

Thus, the prior distribution for the regression coefficient parameters is represented as follows [43]:

$$\left[\pi |\mu_{\pi },\sum_{\pi }\right]=N\left(1_{n\times 1}⊗\mu_{\pi },\sum_{\pi }\right)$$

The vector $\mu_{\pi }={\left(\mu_{\pi 0},\ldots ,\mu_{\pi p}\right)}^{T}$ contains the means of the regression coefficient terms. In addition, the prior on regression coefficient accounts for the possible spatial dependence through the covariance $\sum_{\pi }$. The Bayesian spatial varying coefficient was employed in this study to relax the stationarity assumption. In contrast, the varying coefficient is achieved by specifying the priors for the $\pi^{\prime }s$, and the most adopted model is a simultaneously autoregressive model (SAR) and CAR model. The SAR model is computationally easier to use with likelihood methods.

In contrast, the CAR model is computationally easier for Gibbs sampling used with Bayesian model fitting. Gibbs sampling is an approach that iteratively draws an instance from the distribution of each variable, conditional on the current values of the other variables, to estimate complex joint distributions [43,44]. In the present study, we used the CAR priors for the $\pi^{\prime }s$.

Furthermore, for the specification of the CAR model, we considered $\phi ={\left(\phi_{1},\ldots ,\phi_{k}\right)}^{T}$, with k components that follow a multivariate Gaussian distribution having a mean of zero and *B* as the inverse of the dispersion matrix, such that *B* represents a *k* × *k* symmetric and positive definite matrix. By positive definite matrix, we mean a matrix that is symmetric and whose eigenvalues are positive. Therefore, the density for ϕ is given by

$$\pi \left(\phi \right)={\left(2\lambda \right)}^{-k/2}{\left|B\right|}^{-1/2}\exp\left\{\frac{1}{2}\phi^{T}B\phi \right\}$$

For the CAR model, the conditional distribution of one component in terms of the elements of the matrix $B=a_{ik}$ is expressed as

$$\pi (\phi |\phi_{-i})=exp\left\{\frac{-a_{ii}}{2}{\left(\phi_{i}-\sum_{k=i}\frac{-a_{ik}}{a_{ii}}\phi_{k}\right)}^{2}\right\}$$

This implies that $\phi_{i}|\phi_{-i}\sim N\left(\frac{-a_{ik}}{a_{ii}}\phi_{k},\frac{1}{a_{ii}}\right)$. Let $G=(g_{ik})=\frac{-a_{ik}}{a_{ii}}$ and $H=diag(\tau_{1}^{2},\ldots ,\tau_{i}^{2})$, such that $g_{ik}\tau_{k}^{2}=g_{ki}\tau_{i}^{2}$. Thus, the inverse of the dispersion matrix B is related to G and H as illustrated below:

$$B=H^{-1}\left(I-G\right)$$

The joint distribution ϕ is $MVN\left(0,H^{-1}\left(I-G\right)\right)$, provided $\left(I-H\right)G^{-1}$ is symmetric and positive definite, and *I* is the identity matrix [45]. As reported by [46,47], the logic here is that *G* and *H* must be appropriately modeled to ensure symmetry in *B*, while matrix *G* indicates the relationship between the neighbors. Notably, the CAR model is an attractive way to handle spatial statistical dependencies (see [28,46]). Typically, the prior for the structured and unstructured random effects followed the CAR model and an independently and identically distributed (i.i.d.) normal distribution.

Similarly, the specification of the Bayesian SVC can be completed with the description of the prior distribution. The posterior distribution for a model of this type cannot be handled analytically. Thus, a fully Bayesian integrated method based on INLA implemented in R-INLA, a package built within the R statistical package for approximating the model parameters, was utilized [47,48]. Conclusively, all of the models used in the analysis were compared using the deviance information criterion (DIC) values [49,50]; the model with the smallest DIC values was preferred for estimating the parameters [51,52,53,54]. *DIC* is defined as $DIC=\bar{D}+pD$, where $\bar{D}$ is the posterior mean of the model deviance, which is a measure of goodness of fit, and *pD* is the adequate number of parameters, which indicates the complexity of the model and penalizes over-fitting.

## 3. Results

### 3.1. Descriptive Statistics

The statuses of both hypertension (yes, no) and diabetes (yes, no) were considered as two outcome variables. The predictors introduced in the model were sex (male versus female); age (years); race: African versus White, Indian/Asian, or Colored; working for a wage: yes or no; working without remuneration: yes or no; salary period: per week, monthly, or annually; type of residence: urban or rural; educational status: no primary education versus primary, secondary, or tertiary; and marital status: single versus married, widowed/divorced/separated. Table 1 shows the statistical description of people living with diabetes and hypertension across the covariates. The GHS data come from private households in all nine provinces of South Africa and residents in workers’ hostels. Among the 68,986 people interviewed, 33,151 (48.1%) were male, and 35,835 (51.9%) were female. The racial spread of the data specifies that, out of all of the people interviewed in this survey, the highest proportion (57,930, 84%) were black Africans. In contrast, the lowest proportion (1217, 1.8%) belonged to the Indian/Asian group. Additionally, the GHS 2019 sample included 68,986 people aged zero (0) years and above, but only 35 years or above (5571) were included in the model.

### 3.2. Model Performance Comparison

To assess the effect of various individual-level predictors on hypertension and diabetes, four models described earlier were fitted separately for hypertension and diabetes and compared using their DIC values to identify the best fitted model. The DICs for the stationary and SVC models are presented in Table 2 and Table 3, respectively. The selection of the best model is based on DIC values. Findings revealed that the SVC models had a lower DIC than the stationary models. Additionally, it can be seen from Table 3 that Model 7 had the smallest DIC value for both diabetes and hypertension. Models with differences in DIC values less than 3 cannot be differentiated, while those with differences between 3 and 7 can be weakly differentiated [55]. Thus, further interpretation of the results is based on Model 7.

### 3.3. Spatially Varying Effects of Diabetes

**Figure 2:**
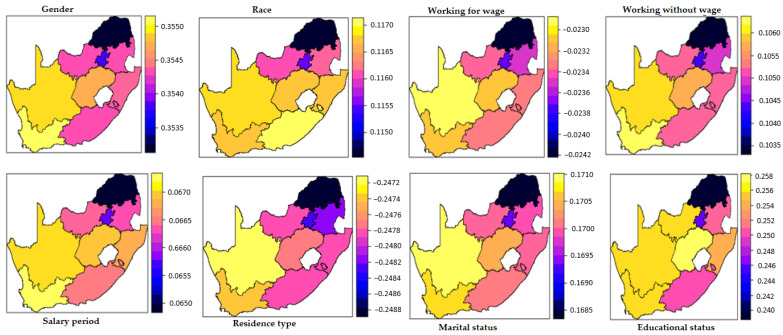
Spatially varying effects of covariates on diabetes.

**Figure 3:**
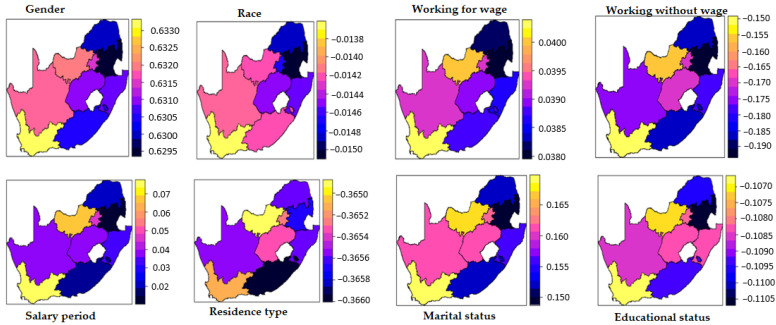
Spatial varying effects of covariates on hypertension.

This national-level study embodied nine provinces of urban and rural areas of South Africa from data collected from the GHS. The entire country entails nine provinces: Eastern Cape, Free State, Gauteng, KwaZulu-Natal, Limpopo, Mpumalanga, Northern Cape, North West, and Western Cape (Figure 1). Choropleth is a map that divides different geographical provinces based on a data variable. It is a type of thematic map where other provinces are shaded according to the covariate under consideration and the proportion of representation of the covariate for a province. The data covariate uses color progression to represent itself in each map province. Thus, a choropleth uses color-coding to indicate quantitative values across geographical areas on a map. The choropleth maps in Figure 2 and Figure 3 suggest that the effects of some of the covariates vary spatially. The DIC value affirms the superiority of the SVC models over their stationary counterparts, particularly the hypertension model. The choropleth maps reveal the varying effects of each covariate through space.

Meanwhile, the maps depicted in Figure 2 indicate that the covariates’ effects in the models vary through space. For instance, considering the varying spatial effect of gender on diabetes, the choropleth shows that the spatial effects for the model fitted ranged from about 0.3535 to 0.3550. Usually, the color scale is darker for the large values, while the lighter color scale is associated with the small ones. Meanwhile, the impact of educational status on people living with diabetes was more pronounced in the North Cape, Western Cape, North West, and Free State, specified by the light yellow to orange shading. Additionally, the effect of working for a wage and without was almost identical across the country, except for some parts of Gauteng province, where the effect was more significant, as specified by blue shading on the choropleth map. Thus, identifying the implications of individual covariates for each province can help to inform measures to curb the diabetes burden.

### 3.4. Spatially Varying Effects for Hypertension

Additionally, as previously explained, the choropleth maps reveal the varying effects of individual covariates through space and indicate that the impact in the models varies. For example, hypertension (shown in blue shading) in those working without a wage was lesser in the Eastern Cape, Limpopo, and KwaZulu-Natal parts than in other provinces. As well, the effect of place of residence also speckled spatially. The effects (yellow shading) were higher in the North West province. On the other hand, the effect of educational status on hypertension was more pronounced (shaded in light blue) in Eastern Cape and Limpopo compared to other provinces. Thus, identifying the implications of individual covariates in each province can help to inform measures to curb the risk of hypertension.

### 3.5. Spatial Effects

**Figure 4:**
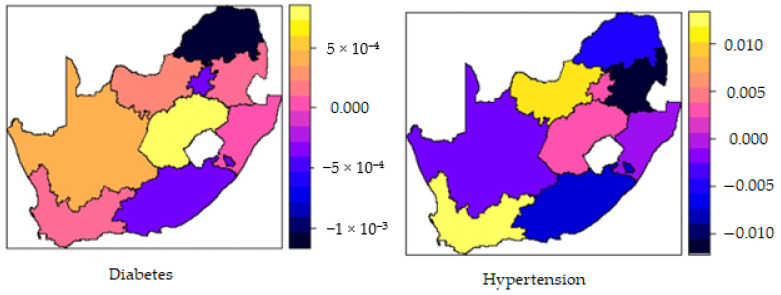
Map of South Africa showing posterior means of spatial effects of diabetes and hypertension.

Figure 4 reveals the results of the spatial effects for the fitted model after controlling for other covariates. Also shown is the 95% posterior probability map of significance. The colors on the choropleth map show the log-odds scale, individually indicating the province’s influence on diabetes and hypertension. Moreover, the choropleth map also revealed a significant spatial effect of 0.001 to 0.0005 (diabetes) and −0.010 to 0.010 (hypertension) (Figure 4). Provinces marked in blue and black had a negative spatial effect and were associated with lower odds of diabetes and hypertension. Provinces shown in yellow and orange had a positive spatial effect and were associated with higher odds of diabetes and hypertension. Spatial effects are surrogates for unknown environmental factors and climate influences.

### 3.6. Non-Linear Effect of Age

**Figure 5:**
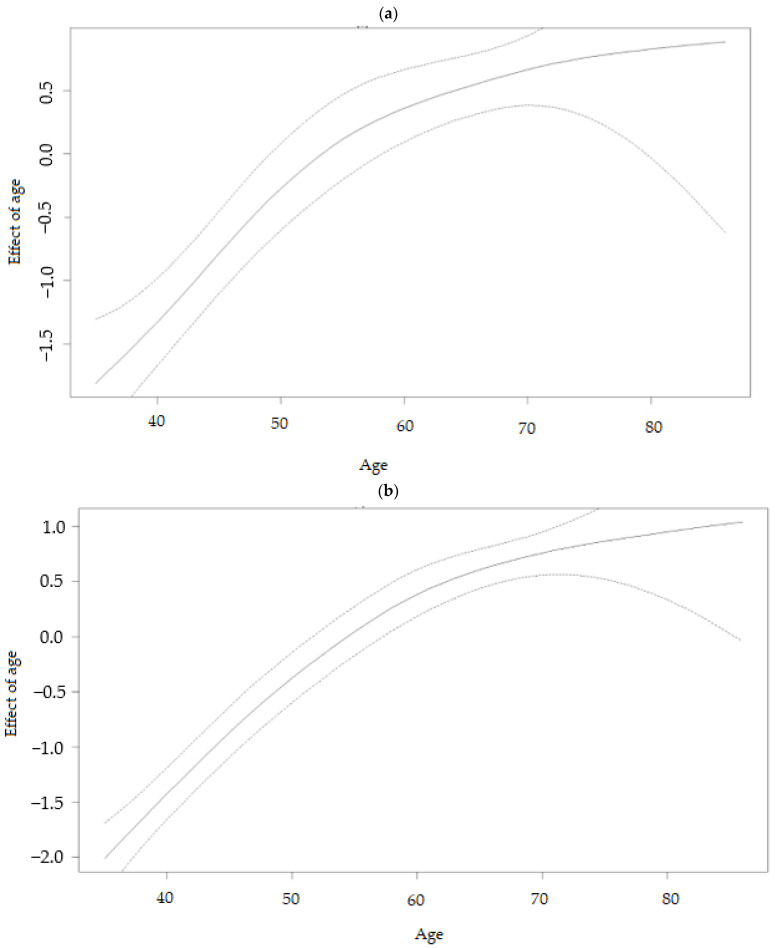
(**a**) Non-linear effects of age on the log-odds of diabetes (posterior means with the 97.5% credible interval). (**b**) Non-linear effects of age on the log-odds of hypertension (posterior means with the 97.5% credible interval).

Another advantage of using the SVC model is incorporating non-linear effects due to continuous covariates. Figure 5a,b gives the posterior mean of the smooth function, estimating the impact of the respondent’s age as a non-linear effect, and its 95% confidence interval. The age of individuals had non-linear implications for diabetes and hypertension, as shown in Figure 5a and Figure 5b, respectively. The solid black lines represent the posterior, while the dashed lines indicate the 80% and 95% credible intervals. It is evident from Figure 5a,b that, as respondent age increased, its effect on diabetes and hypertension also increased. The risk of diabetes and hypertension was lower among respondents 40 to 50 years of age. Figure 5a displays a nearly logarithmic form for respondent age. It was apparent that the effect of respondent age was non-linear.

## 4. Discussion

This study focused on visualizing and assessing spatial patterns of diabetes, hypertension, and socio-demographic features and identified significant spatial variations in the associations between these variables and diabetes and hypertension. These two diseases pose a serious health risk to citizens, and their determining factors relate to varying degrees among individuals and within provincial settings. The 2019 South Africa General Household Survey data were used for the analysis, where a fully Bayesian approach was implemented. Moreover, the non-linear effect of respondent age was modeled using a second-order random walk. To the best of our knowledge, the SVC model proposed for these two diseases had not been investigated previously. We proposed SVC to examine the effects of some selected covariates and non-linear effects on diabetes and hypertension. This study adopted the Bayesian approach because it can produce a reliable and more robust estimation. This study found that the impact of covariates on diabetes and hypertension varied spatially; however, the spatially varying hypertension model differed significantly from the stationary model. The significant benefit of the SVC model is that it can show the effect of each covariate on hypertension in each district [54,56,57]. Additionally, the SVC model allowed the mapping of the residual spatial impact while considering the outcome of the non-linear covariates on the assumption of additivity. This approach allows the assessment of the understated influence of the continuous covariates’ non-linear relationship, which is impossible in a linear model. Moreover, there was a spatial variation in diabetes and hypertension cases among provinces.

The effects of educational status on people living with hypertension were dominant in the Eastern Cape and Limpopo provinces. In contrast, the effect was dominant for people living with diabetes in the North Cape, Western Cape, North West, KwaZulu-Natal, and Free State. Thus, the findings of our study show that educational status has different effects across the provinces of South Africa when investigating NCDs. This regional variation could well be due to differences in lifestyle, level of urbanization, and health care services. Indeed, based on the trend and prevalence of diabetes and hypertension by province, studies revealed that those with less than a high school education had a higher prevalence of hypertension and diabetes in Limpopo than in other provinces [58]. Additionally, other studies have established that education is related to NCDs [59,60]. The effect of marital status on people living with hypertension was dominant in the Eastern Cape, KwaZulu-Natal, and Limpopo provinces. This finding could be ascribed to traditional practices in sub-Saharan African countries, such as wife inheritance. Residential type affected hypertension in the Northern Cape, Limpopo, KwaZulu-Natal, and Mpumalanga provinces.

The respondent’s age was found to have a non-linear relationship with diabetes and hypertension (Figure 5). The risk of diabetes and hypertension was highest among individuals aged up to 70 years, compared to their counterparts between 40 and 50 years. The results show a similar pattern for both diseases examined in this study, implying that both diabetes and hypertension prevalence peak among middle-aged individuals (around age 60 years). Consistent with previous studies, this study showed that the effect of age on diabetes is nearly logarithmic with respect to respondent age [61,62]. Moreover, the study on the prevalence and associated factors of hypertension in a national sample of older South Africans who participated in the Study of Global Ageing and Adults’ Health found high rates of hypertension among older adults (50 years and more) in South Africa [63,64]. Spatial effects in the model account for unobserved variables that correspond to those variables that vary spatially. Thus, not accounting for spatial variability could result in skewed results, biased estimates, and inappropriate decisions on the researcher’s part. Therefore, identifying high prevalence provinces and the relationship between diabetes and hypertension can offer more insight, which can be beneficial in developing policies and strategies for specific provinces. The SVC models proposed in this study were assumed to provide improved smoothing compared to the stationary models, because they give a covariate effect on diabetes and hypertension in each province.

### 4.1. Policy Implications

This study has some policy implications for intervention and program design. First, mapping people living with diabetes and hypertension is essential to assist countries in designing an appropriate mechanism to protect vulnerable people and reduce pressure on health systems. This evidence can also enlighten a comprehensive assessment aimed at protecting many individuals’ social and economic implications. In addition, it is highly imperative for the health sector and regional health to offer care to those hot spot provinces, considering the predictors in order to advance and devise adult-targeted health programs. Meanwhile, detecting all provinces or districts at some risk is imperative for making plans for likely health problems [65,66] and designing active policies to decrease transmission to individuals in a specific location. Statistical modeling implies that it gives policymakers the information needed to make informed decisions in uncertain circumstances. Finally, the choropleth map may assist in measuring the impact of the NCD risk at all regional levels. Further, it can guide in detecting high-risk provinces for targeted interventions and evaluating the effects of intervention plans, which can help policymakers and other public health institutions to map programs targeting these provinces.

### 4.2. Limitations of the Study

The current study has some limitations that must be considered while interpreting the findings outline. First, the set of covariates used was that most often considered in NCD risk evaluations. However, some relevant factors, such as dietary habits, physical exercise, smoking status, biomarker data, and alcohol consumption [12,14], which may increase the risk of hypertension and diabetes, were not captured, due to the unavailability of data. Thus, to implement this methodology for future work, we advocate the inclusion of those relevant available variables. Second, it was a cross-sectional survey; therefore, no causal inferences could be made from the results and findings. Since the study was based on secondary datasets, we were limited to using only the variables found in the GHS. Despite these limitations, the study’s strength lies in the methodology adopted. Similarly, the regression method incorporated a SVC model to capture the non-linearity in our analytic approach, resulting in unbiased estimates. The other robustness of this study was using GHS data, which offered a considerable sample size. 

## 5. Conclusions

In conclusion, there are spatial effects on diabetes and hypertension in South Africa. The results suggest that province-specific factors are most likely to increase the number of cases of diabetes and hypertension. Moreover, this study underlines the vital part that different covariates might play in the spatial variability of people living with diabetes and hypertension in South Africa. Evaluation of province-specific factors of diabetes and hypertension in the province should be necessary. The spatial distribution of these covariates offers improved evidence for producing detailed maps for people living with diabetes and hypertension. These maps illustrate the high spatial variation of people living with diabetes and hypertension at the regional level of South Africa. Considering the one obtained in the current study, a comprehensive mapping of the spatial structure of people living with diabetes and hypertension could improve program efficiency. Such maps make information available to advantageously target regions and inform the most needed policies and strategies for resource allocation. Similarly, another relevant implication evolving from this study is that it would be precious for census data. For instance, census data are regarded as one of a country’s main secondary data sources. It would be interesting to initiate a detailed search on this issue, considering that more respondent background variables are primarily available in census data. Therefore, considering this type of data source (census data), more comprehensive and spatial structures could be revealed, highly germane for analytical and policymaking purposes, since many spatial analyses require detailed demographic information. Finally, this paper contributes to understanding spatial variations in diabetes and hypertension in South Africa by applying a Bayesian SVC model approach based on the INLA technique. More importantly, by proposing this novel approach, we established a regional variation in diabetes and hypertension prevalence within the nine provinces of South Africa. These observations may have public health significance, considering the lack of strategies to prevent and control diabetes and hypertension efficiently. This study is the first to map diabetes and hypertension in South Africa using a household survey, to the best of the authors’ knowledge. The map could have significant implications for the perception of how diabetes and hypertension are spatially distributed and help health promotion programs to allocate the resources efficiently. Moreover, from a public health view, it is essential to map diabetes and hypertension, because this information could enlighten the development of prevention programs on a community level.

## Figures and Tables

**Table 1:** Descriptive summary of variables utilized in the study.

	Description	*n* (Percent)
Metrical Variable		Mean (SD)
Age	Age of the respondent	46.45 ± 8.22
Socio-demographic variables		
Sex		
	Male	2807 (50.4)
	Female	2764 (49.6)
Marital status		
	Single	1701(30.5)
	Married	3203 (57.5)
	Divorced/Separated/Widowed	667 (12.0)
Educational status		
	No primary education	225 (4.0)
	Primary	990 (17.8)
	Secondary	3508 (63.0)
	Tertiary	848 (15.2)
Race		
	African	4718 (84.7)
	Colored	496 (8.9)
	Indian/Asian	57 (1.0)
	White	300 (5.4)
Working for a wage		
	Yes	4547 (81.6)
	No	1024 (18.4)
Working without remuneration		
	Yes	83 (1.5)
	No	5498 (98.5)
Salary period		
	Per week	762 (13.7)
	Per month	4775 (85.7)
	Annually	34 (0.6)
Residence type		
	Urban	3861(69.3)
	Rural	1710 (0.6)
Province		
	Western Cape	489 (8.8)
	Eastern Cape	700 (12.6)
	Northern Cape	380 (6.8)
	Free State	398 (7.1)
	KwaZulu-Natal	548 (9.8)
	North West	387 (6.9)
	Gauteng	1557 (27.9)
	Mpumalanga	600 (10.8)
	Limpopo	512 (9.2)

**Table 2:** Bayesian values of stationary model diagnostic measures.

Outcome	Model Fit Statistics	Model 1	Model 2	Model 3	Model 4
Diabetes	pD	14.78	17.77	17.71	17.65
	$\bar{D}(\theta )$	2166.80	2067.81	2068.02	2068.16
	DIC	2181.58	2085.58	2085.73	2087.37
Hypertension	pD	14.91	24.85	21.67	24.19
	$\bar{D}(\theta )$	4723.10	4382.83	4391.61	4389.19
	DIC	4738.01	4407.68	4413.28	4413.38

**Table 3:** Bayesian values of SVC model diagnostic measures.

Outcome	Model Fit Statistics	Model 5	Model 6	Model 7	Model 8
Diabetes	pD	12.11	12.41	12.92	12.35
	$\bar{D}(\theta )$	2074.84	2074.41	2073.74	2074.49
	DIC	2086.95	2086.82	2086.66	2086.84
Hypertension	pD	16.97	17.26	16.91	17.91
	$\bar{D}(\theta )$	4385.94	4386.39	4383.82	4388.54
	DIC	4402.91	4403.65	4400.73	4406.45

## Data Availability

The data presented in this study are available on request from the corresponding author.
